# Development of a Machine Learning Based Web Application for Early Diagnosis of COVID-19 Based on Symptoms

**DOI:** 10.3390/diagnostics12040821

**Published:** 2022-03-27

**Authors:** Charlyn Nayve Villavicencio, Julio Jerison Macrohon, Xavier Alphonse Inbaraj, Jyh-Horng Jeng, Jer-Guang Hsieh

**Affiliations:** 1 Department of Information Engineering, I-Shou University, Kaohsiung City 84001, Taiwan; isu10903050d@cloud.isu.edu.tw (J.J.M.); xalphonse@gmail.com (X.A.I.); jjeng@isu.edu.tw (J.-H.J.); 2 College of Information and Communications Technology, Bulacan State University, Malolos City 3000, Philippines; 3 Department of Electrical Engineering, I-Shou University, Kaohsiung City 84001, Taiwan; jghsieh@gmail.com

**Keywords:** COVID-19, COVID-19 symptoms, disease detection, machine learning algorithms, hyperparameter optimization, cross-validation, online disease diagnosis, online symptom checker, web application

## Abstract

Detecting the presence of a disease requires laboratory tests, testing kits, and devices; however, these were not always available on hand. This study proposes a new approach in disease detection using machine learning algorithms by analyzing symptoms experienced by a person without requiring laboratory tests. Six supervised machine learning algorithms such as J48 decision tree, random forest, support vector machine, k-nearest neighbors, naïve Bayes algorithms, and artificial neural networks were applied in the “COVID-19 Symptoms and Presence Dataset” from Kaggle. Through hyperparameter optimization and 10-fold cross validation, we attained the highest possible performance of each algorithm. A comparative analysis was performed according to accuracy, sensitivity, specificity, and area under the ROC curve. Results show that random forest, support vector machine, k-nearest neighbors, and artificial neural networks outweighed other algorithms by attaining 98.84% accuracy, 100% sensitivity, 98.79% specificity, and 98.84% area under the ROC curve. Finally, we developed a web application that will allow users to select symptoms currently being experienced, and use it to predict the presence of COVID-19 through the developed prediction model. Based on this mechanism, the proposed method can effectively predict the presence or absence of COVID-19 in a person immediately without using laboratory tests, kits, and devices in a real-time manner.

## 1. Introduction

A global pandemic called coronavirus disease (COVID-19) is a contagious disease caused by SARS-CoV-2 virus. COVID-19 carriers will experience mild to moderate respiratory illness, while some may experience serious symptoms that requires immediate medical attention, especially older people with underlying comorbidities [1]. What is alarming about COVID-19 is that anyone who gets infected with this virus may be seriously ill or die at any age. However, there were many ways to prevent and slow down COVID-19 transmission. According to the World Health Organization (WHO), getting vaccinated, practicing social distancing for at least one meter, wearing masks properly, staying in well-ventilated places, frequent hand washing, using alcohols, and covering your nose and mouth when coughing or sneezing can prevent an individual from getting infected [1]. As of 16 February 2022, there were 416,014,373 confirmed COVID-19 cases and 5,856,697 reported deaths worldwide [2]. Confirmed cases increased by almost 100% in just a span of 5 months as reported last 30 September 2021 with 234,057,967 COVID-19 cases [3].

COVID-19 has mutated into several variants, and the Centers for Disease Control and Prevention (CDC) recorded two variants of concern (VOC). VOC refers to a variant that has evidence of increased transmissibility and severity of the disease, significant reduction in neutralization against a person’s antibodies, reduced effectivity of treatments or vaccines, or failures in the diagnosis [4]. A VOC requires more appropriate and immediate public health actions as its outbreak will cause danger to human lives. The first VOC was the delta variant as labeled by the WHO, which was first identified in India [4]. The delta variant has evidence of increased transmissibility and resistance to the developed vaccines. The second VOC was labeled the omicron variant, which was first identified in South Africa with potential increase of the virus transmissibility as well as reduction in neutralization by antibodies and vaccines [4].

Since COVID-19 is a contagious disease and has mutated into several variants that reported increased transmissibility, it is necessary to prioritize its early detection. Through early diagnosis of the virus, isolation can be immediately imposed to prevent the spread of the virus. A COVID-19 positive person can be identified through the use of testing kits such as COVID-19 real-time reverse transcription–polymerase chain reaction (RT-PCR) and antigen testing. However, these testing kits have costs and are not always available on hand. Due to the virus outbreak, limited access to COVID-19 testing kits hinder the early diagnosis of the disease [3]. In line with this, an accessible and real-time COVID-19 prediction model is necessary.

This study focuses on developing a machine learning-based web application for the early diagnosis of COVID-19. Many research attempts utilizing machine learning algorithms to detect the presence of COVID-19 e were published, and we devised a list in Table 1.

Based on previous researches, it is evident that machine learning can be utilized to detect the presence of COVID-19, reduce the spread of the virus, predict cases, minimize the death counts, and most of all, take over some of the workload of doctors and nurses during the pandemic. Predictors utilized to detect COVID-19 presence were laboratory findings, blood test results, X-ray images, and CT Images; however, none has focused specifically on detecting COVID-19 presence using symptoms without the need of laboratory tests. Since COVID-19 demands early diagnosis to prevent the transmission to other people, a COVID-19 prediction model that will allow users to provide the current symptoms being experienced will be proposed in this study. In this way, the potential beneficiaries of this prediction model can be notified with immediate results as to whether they are COVID-19 positive or negative. This application does not require any laboratory tests, and it is accessible anywhere even at home as long as the user is connected to the internet. The model will be developed by applying a supervised machine learning algorithm through analyzing COVID-19 symptoms using the Python programming language. Google colab research (Mountain View, CA, USA) was used in developing the model, and the developed model was integrated in the web application using the Django Framework (Django Software Foundation, Atlanta, GA, USA). The source codes and the prediction model will be deployed using the Github repository (San Francisco, CA, USA) and Azure Web Services (Microsoft, Redmond, WA, USA) to make it available for public use.

The rest of this study was organized as follows: Section 2 discusses the methodology of this study including the machine learning modeling steps. Section 3 is the results and discussion section, which briefly explains the development process of a machine learning-based web application integrating the developed model and its workflow. Section 3 also discusses the overview on how to use the web application, and the deployment of the project to make it accessible online. Lastly, Section 4 is the conclusions section.

## 2. Materials and Methods

**Figure 1:**
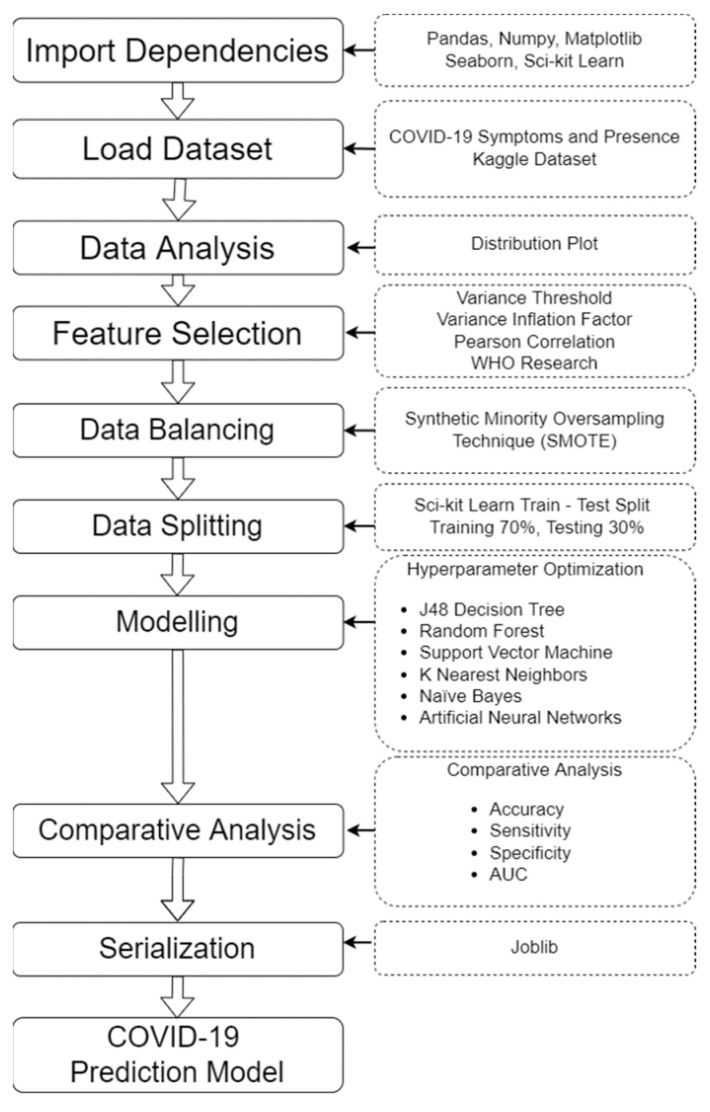
The Block Diagram of the process of machine learning modeling.

The machine learning modeling process was performed using Google colab research, which is a platform used to write and execute Python codes, with free access to a Graphics Processing Unit (GPU) that is well suited for data science [11]. The process of machine learning modeling can be seen in the block diagram shown in Figure 1.

In Figure 1, the 10 phases of the development of a COVID-19 prediction model were displayed. The steps were importing dependencies, loading the dataset, data analysis, feature selection, data balancing, data splitting, modeling, comparative analysis, serialization, and finally, the developed prediction model to determine the presence of COVID-19.

### 2.1. Importing Dependencies and Loading the Dataset

We used the pandas, numerical python (Numpy), matplotlib, sea-born, and sci-kit learn (sklearn) packages in developing the model. The Pandas package is a fast and easy data manipulation tool that can be used in data analysis, from reading the data from the spreadsheet to devising data frames for facilitating the presentation of the data [12]. Together with Pandas, the Numpy package was also used to perform mathematical and scientific calculations as well as perform high-level mathematical functions on its multi-dimensional arrays and matrices [13]. The data distribution of every column in the dataset must be performed to give a glimpse on how the data are distributed throughout the dataset, and each column must be analyzed according its relationship with random attributes present in the dataset. The data distribution and the correlation coefficient plot can be successfully made through importing the Matplotlib package, particularly the pyplot function, which is intended to perform interactive and programmatic plot generation in a MATLAB-like way [14]. To provide attractive and informative data visualization graphs [15], the Seaborn package was imported, particularly the distplot and heatmap functions. Lastly, the sklearn package was imported since it supports both supervised and unsupervised machine learning by providing various tools for splitting the dataset, model selection, model’s performance evaluation by computing statistical measures, and many other useful functions [16].

Importing the dependencies were required to perform every process included in developing the model such as loading the dataset. For the data collection, we utilized a dataset available from Kaggle entitled “COVID-19 Symptoms and Presence”. There were 21 attributes in the dataset wherein 20 were the possible factors related to acquiring the virus, and the remaining 1 attribute determines the presence or absence of COVID-19 in the sample. The dataset has a total of 5434 rows. The attributes and the descriptions of the dataset are displayed in Table 2.

In Table 2, the predictors and its description are presented. This is a publicly available dataset and the sources are the World Health Organization (WHO) Coronavirus Symptoms and the All India Institute of Medical Sciences (AIIMS).

### 2.2. Data Analysis and Feature Selection

**Figure 2:**
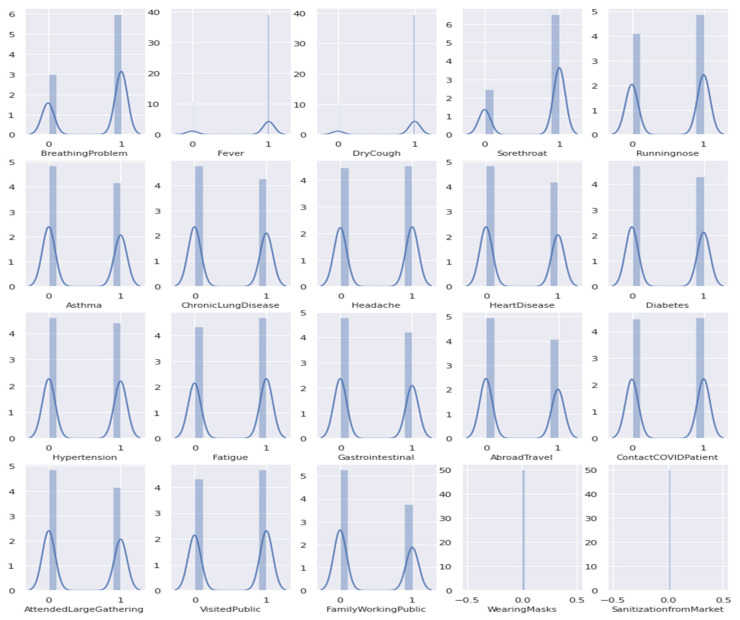
The distplot of the collected dataset.

To be able to depict the distribution of data across the whole dataset, the displot function of the Seaborn package was used along with the Matplotlib. This function plots the data by a histogram combined with a line on it, representing the univariate distribution of a variable against the density distribution [17]. The distplot of the collected dataset can be seen in Figure 2.

In Figure 2, 20 columns in the dataset were presented, which were referred to as the COVID-19-related symptoms, namely breathing problems, fever, dry cough, sore throat, runny nose, asthma, chronic lung disease, headache, heart disease, diabetes, hypertension, fatigue, gastrointestinal, abroad travel, contact with a COVID-19 patient, attended a large gathering, visited public areas, family working in public areas, wearing masks, and sanitization of things bought from the market. The vertical labels (*y*-axis) represent the density of the samples in the dataset, while the horizontal labels (*x*-axis) represent the classes wherein 0 or the indicated symptom is not present in the person, while number 1 means yes or currently being experienced by the person. Based on the distribution plot, a preliminary intuition can be drawn such as the majority of the samples in the dataset were having breathing problems, fever, dry cough, sore throat, runny nose, headache, fatigue, and visited public areas. Meanwhile, some samples in the dataset do not have asthma, chronic lung disease, heart disease, diabetes, hypertension, gastrointestinal, abroad travel history, attended a large gathering, and family working in public areas. Dataset samples have a slightly close density of having contact with COVID-19 patients, while wearing masks and sanitization from the market have only one content that is 0 or no.

We used the variance threshold to perform feature selection to remove the attribute values that do not have significant variance or have the same value for all samples [18]. We used 80% as the threshold and found out that the attributes wearing masks and sanitization from the market exceeded the threshold, since the values were the same for all samples. These columns were removed from the dataset having only 18 features left.

Another feature selection method, which is the Pearson Correlation Coefficient (PCC), was applied in the dataset. This is a statistical measure to determine the correlation of two random attributes [19]. The formula used in this method can be seen in Equation (1).

(1) $$r=\frac{\sum \left(x_{i}-\bar{x}\right)\left(y-\bar{y}\right)}{\sqrt{\sum {\left(x_{i}-\bar{x}\right)}^{2}\sum {\left(y-\bar{y}\right)}^{2}}}$$

where *r* is the Pearson correlation coefficient, $x_{i}$ is the variable’s samples, $\bar{x}$ is the sample mean, $y_{i}$ is the samples of another variable, and $\bar{y}$ is the value of its sample. The value of *r* ranges from −1 to +1. PCC was applied to measure the correlation of the attributes to the target variable, which is the COVID-19 attribute and to know which features were positively and negatively correlated to the target class. By doing this, we can have intuition on what features must be retained in training the model, and the results are displayed in Table 3.

In Table 3, the correlation values of each feature toward the target variable were listed. The highest correlation goes to the symptom sore throat having 0.503 correlation, next is the Dry Cough with 0.464 correlation value, and the Abroad Travel and Breathing Problems have a 0.444 correlation value. Having a positive correlation means that the variables were positively correlated: as *x* increases, the value of *y* also increases, and vice versa. In contrast, the negative correlation means that the variables were negatively correlated: when the value of *x* decreases, the value of *y* increases, and vice versa [20]. Symptoms that were found to be negatively correlated to the target variable were gastrointestinal, runny nose, headache, fatigue, and chronic lung disease. Features that have very low correlation to the COVID-19 variable were asthma, diabetes, and heart disease, which were mostly were termed as comorbidities.

After studying the correlation of the predictors to the target, we also took into consideration the collinearity. Collinearity happens when two predictors are linearly associated or having a high correlation to each other, and both were used as predictors of the target variable [21]. Multicollinearity may also happen, which is a situation wherein the variable has collinearity with more than one predictors in the dataset. We used the Variance Inflation Factor (VIF) to detect the collinearity of the predictors in the dataset. The VIF starts from 1 to infinity, and the value of 1 means that the features were not correlated. VIF values less than 5 are moderately correlated, while VIF values of 10 and above are highly correlated and a cause of concern [21]. The VIF values of each predictor in the dataset can be seen in Table 4.

Table 4 displays the VIF of each predictor in the dataset. The highest is dry cough at 5.38, which is not surprising, since it is one of the most common symptoms of COVID-19 [22]. Predictors such as fever, sore throat, and breathing problems attained the next highest VIF having scores lower than 5. A VIF of 1 to 5 means that the predictors were not correlated and can be considered in building the COVID-19 prediction model. However, we still consider including the dry cough as a predictor in the building of the COVID-19 prediction model even if it has a VIF of 5.38, since this symptom can contribute to predicting COVID-19 in a person, and it is included in the most common symptoms declared by the World Health Organization (WHO).

Aside from the variance threshold, PCC, and VIF, we also used the WHO website to determine the common symptoms of the disease because it has been validated by the experts in the medical field and is updated regularly. Table 5 shows the most common, less common, and serious symptoms of COVID-19.

Table 5 displays the list of symptoms from the WHO’s website. According to the WHO, people who suffer from serious symptoms may cause danger to human lives, so it is necessary to go the nearest hospital to seek immediate medical attention. People who experience mild symptoms but are still healthy may manage themselves at home [22]. It is necessary to undergo self-isolation immediately to prevent the virus from spreading and to prevent possible transmission until tested COVID-19 negative through a Polymerase Chain Reaction (PCR) test.

Based on the findings in the feature selection process, the feature combination that can be used in building the prediction model was devised, which is composed of the positive correlation features. Negatively correlated symptoms were also included but with respect to the symptoms declared by the WHO. The features that will be included in the training process were the sore throat, dry cough, abroad travel, breathing problems, attended a large gathering, contact with COVID-19 patient, fever, family working in public, visited public exposed places, hypertension, asthma, diabetes, heart disease, runny nose, headache, and fatigue.

### 2.3. Data Balancing and Dataset Splitting

**Figure 3:**
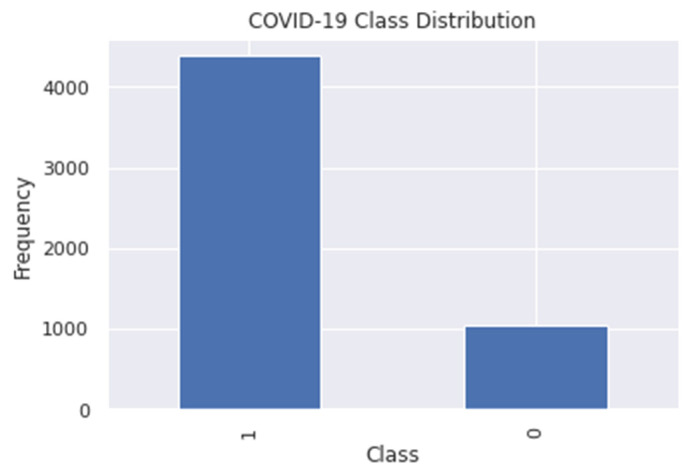
Bar plot of COVID-19 variable class distribution in the raw dataset.

In preprocessing the dataset, we used data balancing, which is important to promote a balanced prediction rate. Dataset splitting was utilized to divide the samples into training and testing datasets. For the COVID-19 Symptoms and Presence dataset, the classes have a 4:1 class imbalance [3], and to address this, we make use of a data balancing technique named “Synthetic Minority Oversampling Technique” (SMOTE) proposed by Chawla et al. in 2002 to perform oversampling in the minority dataset. SMOTE generates additional instances for the minority group by generating additional synthetic samples based on a selected number of neighbors of a random sample [23]. By doing this, the class with fewer samples in the dataset will be increased. It is necessary to balance the dataset to obtain a high accuracy rate, very low error rate, and to avoid classification bias. A bar plot representing the class distribution of the target variable can be seen in Figure 3.

**Figure 4:**
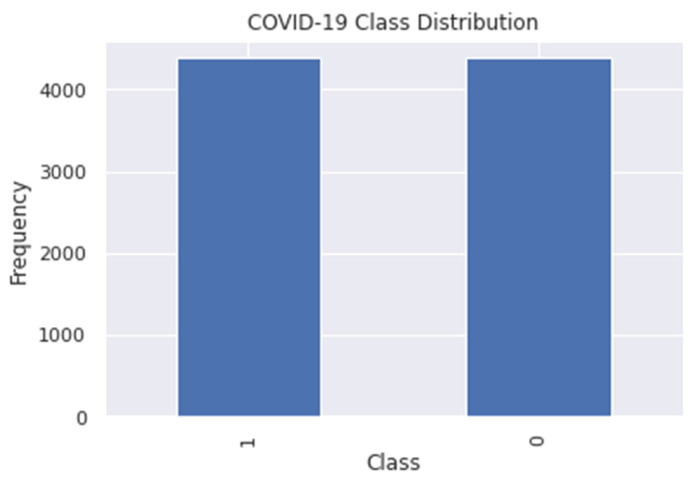
Bar plot of COVID-19 variable class distribution in the balanced dataset.

In Figure 3, the COVID-19 variable class distribution was presented according to the number of samples in the raw dataset collected from Kaggle. The vertical labels indicate the frequency or the number of the samples, while the horizontal label 1 implies the COVID-19 positive samples, and the label 0 indicates COVID-19 negative samples. The total number of samples in the dataset is 5434, where COVID-19 positive samples were 4383 and COVID-19 negative samples were 1051. This kind of dataset will cause prediction bias because a lot of samples were in the COVID-19 positive class, making it well known to the classifier, and there is a high chance that the majority of the data may be predicted as COVID-19 positive. To address this, we used the available imbalanced-learn python package, which offers several re-sampling techniques that can be applied in datasets showing strong data imbalance [24]. This package must be installed in the Google colab research notebook; then, from its oversampling module, the SMOTE function was imported and was applied to the dataset, and the result is presented in Figure 4.

In Figure 4, the dataset is presented in a balanced number of samples as a result of the SMOTE function. The total number of samples in the dataset after applying SMOTE became 8766 where 4383 still belongs to the COVID-19 positive class, and the remaining 4383 samples belong to the COVID-19 negative class. Now that the dataset is balanced, we divided the dataset using a ratio of 7:3; 70% of the samples will be used as the training dataset to develop the COVID-19 prediction model, and the remaining 30% will be used for testing the performance of the model. The data-splitting process was performed using the “train_test_split” function from the sklearn’s “model_selection” module, which randomly splits the given samples, arrays, and matrices into training and testing subsets [16].

By applying the train_test_split function, 6136 samples were included in the training dataset, and the remaining 2630 samples were taken to be used as the testing dataset, which makes the dataset ready for the next process, which is the modeling. We used the 10-fold cross-validation resampling method for all the experiments.

### 2.4. Modeling of the COVID-19 Prediction Model Using Supervised Machine Learning Algorithms

The modeling phase of this study discusses the process of how to develop a COVID-19 prediction model including the hyperparameter optimization, training, testing and evaluation. After data processing using the variance threshold, PCC, VIF, feature selection techniques, and finally, the SMOTE data balancing technique, several models were built using the Google colab research utilizing different supervised machine learning algorithms, namely, J48 DT, RF, SVM, k-NN, NB, and ANN.

1. J48 Decision Tree

The first algorithm we included in this study is the DT, which is an algorithm that creates a tree-like plot wherein nodes represent a condition for any classification task. A generated tree starts with a root node that tests a given sample; an example of a test condition is whether the person has a dry cough or not. After testing the samples using a condition, branches will be generated that separate the samples into their respective classes. J48 DT is a kind of decision tree algorithm primarily utilized in classification tasks.

To attain the highest possible performance of the J48 DT algorithm, we performed hyperparameter tuning using GridSearchCV and 10-fold cross validation for all the experiments. The following hyperparameters were tuned: For the maximum depth of the decision tree, we used the values 2, 3, 5, 10, and 20. In terms of the minimum samples required to be in the leaf node, we used 5, 10, 20, 50, and 100. Lastly, to measure the information gain or the purity of the nodes of the decision tree, the criterion we used were the Gini index and entropy. An innovation of this algorithm was made using several decision trees known as random forest.

2. Random Forest

The RF algorithm provides a significant amount of improvement in the classification accuracy of a model because it is capable of producing multiple decision trees. Each decision tree will generate a result about the sample, and the final result will be generated according to the results of the majority of the decision trees [25]. To obtain the highest possible performance of the RF algorithm, we performed hyperparameter tuning using GridSearchCV and 10-fold cross validation for all the experiments. We also tuned same set of hyperparameters as what we tuned in the DT wherein the maximum depth values of the decision trees to be created are 2, 3, 5, 10, and 20, and the minimum samples required to be in the leaf node, we used 5, 10, 20, 50, and 100. To measure the quality of the split of each node, the criterion we used were the Gini index and entropy. Since the RF algorithm produces several decision trees, we used the number of estimators to set how many trees to be created in the forest. The values used for this hyperparameter were 100, 200, and 300. Each decision tree learns from random sets of samples from the dataset and using bootstrap means that the samples were drawn with replacement [26]. The hyperparameter bootstrap was also tuned with the values either True or False; the entire dataset was used to build a decision tree if the bootstrap parameter was set to False. The next algorithm used in this study is the SVM.

3. Support Vector Machine

Generating hyperplanes is a significant part of the SVM algorithm. Hyperplanes are utilized by SVM in separating the samples in the dataset according to their respective classes. SVM prioritizes in maximizing the distance of each group to the dividing hyperplane. Through the use of hyperplanes, we can minimize the errors in separating the instances. [27]. The kernels used in the experiment were the linear, radial, polynomial, and sigmoid kernels; these kernels will help the SVM algorithm determine which are the best hyperplanes that can separate the dataset into COVID-19 positive and negative classes. A mathematical trick called the “Kernel trick” allows the SVM to create a higher dimensional space from a low-dimensional space dataset; specifically, a single-dimensional data will be converted into two-dimensional data according to their respective classes [28].

A radial basis function (RBF) is one of the most popular among all the kernels, for it can be used to separate datasets that are not linearly separable by adding curves or bumps in the data points. Next is the polynomial kernel function, whose result depends on the direction of the two vectors, and it is the only kernel that has the hyperparameter named “degree”, which determines the flexibility of the decision boundary [29]. The sigmoid kernel which is a quite popular kernel that originated in the neural networks field was also included. The selection of kernel to be used in the classifier depends on the kind of classification to be done; it is not fixed [28]. In line with this, we included linear, radial, polynomial, and sigmoid kernels in the hyperparameter optimization process to find out which kernel will perform better.

The regularization parameter C, which is a hyperparameter common to all the kernels that regulates the misclassification of the samples and the simplicity of the decision boundaries, was tuned. The C values used in the hyperparameter optimization were 0, 0.01, 0.5, 0.1, 1, 2, 5, and 10. The gamma was also considered, which determines how much influence a training sample has. The values used for the gamma were 1, 0.1, 0.01, and 0.001. Large values of gamma means that the other examples are closer to be affected [30]. Another supervised machine learning algorithm used is the k-NN, which uses a number of neighbors or samples surrounding a random sample in the dataset.

4. K-Nearest Neighbors

k-NN uses a sample’s nearest neighbors in the dataset to determine to which class it belongs. k-NN is an old and simple supervised machine learning algorithm used to solve classification tasks [31]. To determine a sample’s nearest neighbor, k-NN uses distance metrics, which are Euclidean and Manhattan distance. The most commonly used distance metric of k-NN is the Euclidean distance, which can be expressed in Equation (2).

(2) $$d\left(x_{i},x_{j}\right)=\sqrt{\sum_{r=1}^{n}w_{r}{\left(a_{r}\left(x_{i}\right)-a_{r}\left(x_{j}\right)\right)}^{2}}$$

where *x* = (*a*_1_, *a*_2_, *a*_3_… *a*_*n*_) is a sample in the dataset in a vector format, the number of the sample’s attributes is expressed as *n*, $a_{r}$ is the *r*^th^ attribute, the weight is expressed as $w_{r}$, and $x_{i}$ and $x_{j}$ are the two samples. Another distance metric used is the Manhattan distance. The Manhattan distance is also called “Taxicab Geometry” and “City Block Distance” [32]. A Minkowski distance formula is used to find the Manhattan distance that works by calculating the distance between the samples in a grid-like path.

Hyperparameter optimization was used to determine the k-NN algorithm configuration that will yield the best possible performance of the model. The total number of neighbors used in the hyperparameter optimization were 3, 5, 7, 9, 11, and 13. The weights hyperparameters used were the uniform and distance. Uniform weights means that all the points in each neighborhood of the sample are weighted equally, while the distance weight allocates points by the inverse of their distance. The closer neighbors of the sample will have more influence than the other neighbors, which are more distant [33]. The next supervised machine learning algorithm used in this study is the naïve Bayes which is based on Bayes’ theorem.

5. Naïve Bayes

NB is another supervised machine learning algorithm based on the Bayes theorem by Thomas Bayes, an English mathematician, which was created in 1763 [34]. The NB algorithm is called naïve because it does not depend on the existence of other parameters; its formula can be expressed in Equation (3).

(3) $$P\left(A|B\right)=\frac{P\left(B|A\right)\;P\left(A\right)}{P\left(B\right)}$$

In Equation (3), the priori probability is expressed as *P*(*A*), which means the probability of event *A* happening. The marginal probability is expressed as *P*(*B*), which means the probability of the event *B* happening. Then, the expression *P*(*A*|*B*) means the posterior probability or probability of *A* happening given that *B* has occurred. The expression *P*(*B*|*A*) means the likelihood probability, which is the probability of *B* happening given that *A* is true [35]. Dividing the product of the likelihood and priori probability by the marginal probability will determine the posterior probability [35].

We used the GausianNB, which is a special kind of NB algorithm, and tuned its only parameter, the variance smoothing. Variance smoothing is another form of Laplace smoothing, which is a technique that helps in the problem of zero probability in the NB. In using high values of alpha, it will push the likelihood toward a value of 0.5 [36]. We used np.logspace(0, −9, num = 100) as the var_smothing value in the GridSearchCV hyperparameter tuning function.

6. Artificial Neural Network

**Figure 5:**
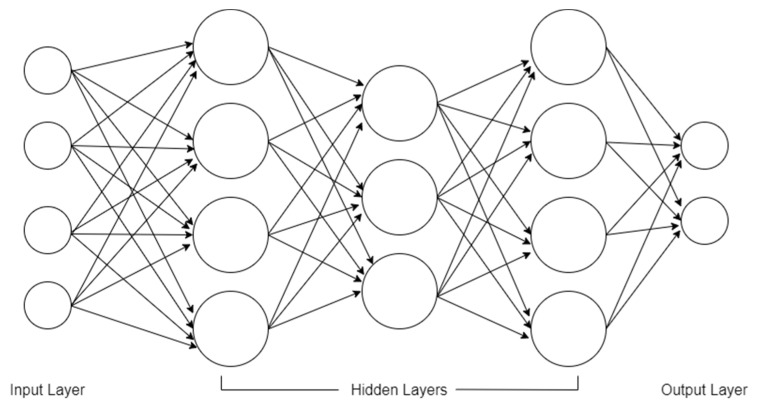
A sample topology of multilayer perceptron with 4 inputs, 3 hidden layers with size of 4, 3, and 4 neurons, respectively, and 2 neurons as the output layer [37].

The last machine learning algorithm we used is the multilayer perceptron (MLP) artificial neural network, which is one of the most commonly used architectures of neural network because of its versatility in classification and regression problems [37]. We plotted an illustration of MLP including the input layer, hidden layer, and the output layer; it can be seen in Figure 5.

In Figure 5, the first and the last layer are called the input layer and the output layer, respectively. The input layer introduces the MLP to the predictors given in the dataset. The output layer carries the final classification result as computed and processed by the hidden layers. The layers in between are called the hidden layers, where all the data processing and the classification of the predictors take place. Commonly, two hidden layers is enough to perform a classification task, but additional hidden layers are recommended to solve more complicated classifications and to discover deeper patterns from the predictors.

We tuned the following hyperparameters to attain the highest possible performance of the MLP ANN. As for the hidden layer sizes, first, there are 3 hidden layers with the size of 50, 50, 50; next, there are 3 hidden layers of 50, 100, and 50 sizes, and lastly, there is 1 hidden layer with the size of 100 neurons. For the activation function of the hidden layer, the “tanh” or hyperbolic tan function and relu rectified linear unit or “relu” were used. For weight optimization solver, we used “sgd” or the stochastic gradient descent. In addition, the solver adam was used, which is the default solver in the MLP classifier of sci-kit learn, which performs well in the training and validation of large datasets. Alpha values are 0.0001 and 0.05. Lastly, the learning rate is either constant or adaptive.

There are a few methods to fine-tune parameters, e.g., grid search, random search, and Bayesian optimization. Grid search requires defined values of the hyperparameters to be evaluated, which performs generally well especially in spot checking combinations [38]. Random search defines a search space wherein random points will be evaluated; it performs great in discovering good hyperparameters combinations not particularly guessed by the developers; hence, it requires more time to execute [38]. Lastly, the Bayesian optimization algorithm is used for more complex optimization problems such as global optimization that finds an input that determines the lowest or the highest cost of a given objective function [39].

We aim to discover the best configuration of the supervised machine learning algorithms in developing the COVID-19 prediction model using defined values of the hyperparameters, and with that, the grid search method was selected. We make use of the GridSearchCV function from sklearn’s model_selection package. GridSearchCV will traverse through the defined hyperparameter values and use those configurations to fit the classifier onto the training set [40]. The hyperparameter optimization will begin by creating a dictionary of the hyperparameters to be tuned including the desired values of it. Then, the GridSearchCV function will execute all the combinations of the given values and evaluate it using cross-validation. In this study, we used 10-fold cross-validation for all the experiments.

The results of this process were the accuracy or loss of each combination, and from there, we can choose the hyperparameter combination that will bring out the best possible performance of the algorithm for both the training and testing dataset [40].

### 2.5. Comparative Analysis Serialization and COVID-19 Prediction Model

Accuracy alone is not enough to choose the best model to be used, and other performance results of the model must be taken into consideration [41]. Moreover, accuracy works best if the dataset is symmetric or has close or equal counts of samples per class [42]. In lieu of comparative analysis, the performance measures used in finding the model that will serve as the most appropriate machine learning algorithm to be used in building a COVID-19 prediction model are as follows.

#### 2.5.1. Performance Criteria

We performed a comparative analysis of the performances of different supervised machine learning algorithms using 10-fold cross-validation, and the important criteria used in this phase are the following:

1. Accuracy

Accuracy is the measurement of all the correctly predicted instances over the total predictions made by the model [3]. It computes the ratio of the correctly classified samples, which are true positives (*TP*) and true negatives (*TN*), over the total number of predictions, which includes the *TP*, *TN*, and misclassified predictions such as false positives (*FP*) and false negatives (*FN*). The formula for accuracy can be seen in Equation (4).

(4) $$Accuracy=\frac{TP+TN}{TP+TN+FP+FN}$$

2. Sensitivity

Sensitivity is the ratio of correctly classified COVID-19 positive samples to all of the COVID-19 positive samples in the dataset. The sensitivity of the classifier is also known as the True Positive Rate (*TPR*), which can be computed using Equation (5) [43].

(5) $$Sensitivity=\frac{TP}{TP+FN}$$

3. Specificity

Specificity is the proportion of the COVID-19 negative cases that were correctly classified as COVID-19 negative, which is also known as the True Negative Rate (*TNR*). The specificity score can be computed using the following formula shown in Equation (6) [43].

(6) $$Specificity=\frac{TN}{TN+FP}$$

Specificity is the inverse of the False Positive Rate (*FPR*), which can be computed using Equation (7) [44].

(7) $$FPR=1-Specificity\;=\frac{FP}{TN+FP}$$

4. AUC

In computing the AUC, the Receiver Operating Characteristics (ROC) curve must be devised first, which is a graphical representation of the model’s performance with respect to various thresholds used in classifying the samples [44]. By analyzing the ROC curve, the model’s capability to classify the samples will be determined. It is plotted using the sensitivity or the TPR on the *y*-axis against the FPR, which is plotted on the *x*-axis. A higher AUC means that the developed COVID-19 prediction model can successfully predict COVID-19 positive samples as positive and COVID-19 negative samples as negative.

#### 2.5.2. Hyperparameter Optimization, Comparative Analysis, and Serialization

This study aims to compare supervised machine learning algorithms to determine which is the most appropriate algorithm to be used in developing a COVID-19 prediction model; however, the optimal performance of an algorithm can be achieved if the best configuration has been utilized in the modeling process. As a result of this, we performed hyperparameter optimization to determine the values at which the algorithm will perform best using the COVID-19 Presence and Symptoms dataset. We utilized the GridSearchCV method using 10-fold cross validation. For the J48 DT algorithm, we tuned the criterion, minimum samples of leaf, and maximum depth hyperparameters. The results are displayed in Table 6.

In Table 6, 10 experimental results for the hyperparameter optimization of J48 DT were displayed, describing the values for the hyperparameters criterion, minimum samples in the node, maximum depth hyperparameters, the attained accuracy for each combination, and the ranking. Based on the results of J48 hyperparameter optimization, we found 2 hyperparameter combinations that attained the highest accuracy, which is 98.52. The first combination is Gini criterion, a minimum of 20 samples in the node and a maximum depth of 5. The next hyperparameter combination is Gini criterion, a minimum of 10 samples in the node, and a maximum depth of 5 as well. The criterion entropy also yielded high scores around 97–98. In this study, the configuration defined in the row in bold format was utilized in building the COVID-19 prediction model. The same condition was applied for the rest of the tables.

The next algorithm used was the RF algorithm; we tuned the criterion, max depth, minimum samples in the node, number of estimators, and bootstrap hyperparameters. The results of the hyperparameter optimization of the RF algorithm are displayed in Table 7.

In Table 7, 10 experimental results for hyperparameter optimization of RF are displayed, describing the values for the criterion, maximum depth, minimum samples in the node, the number of estimators, and bootstrap hyperparameters. Table 7 also displays the attained accuracy for each combination and the ranking. For RF, we found out that the criterion Gini, maximum depth of 20, minimum of 5 samples in the node, 200 trees in the forest, and no bootstrapping will attain the highest accuracy of 98.75. The RF accuracy is 0.23 higher than the J4 DT algorithm in this experiment.

The next algorithm tested was the SVM, and we tuned the regularization parameter C, degree, gamma, and kernel hyperparameters. The results of hyperparameter optimization for the SVM algorithm are shown in Table 8.

In Table 8, 10 experimental results for the hyperparameter optimization of SVM were displayed, describing the values for the C, degree, gamma, kernel, the attained accuracy, and the ranking. For SVM, we found out that the first 10 high-performing classifiers were attained using various hyperparameter combinations, for the hyperparameter C, 1, 2, 5, and 10, and for the degrees 2 and 3. For the kernels hyperparameter, the polynomial and radial basis function yielded the best possible performance of the classifier. As reflected in Table 8, all the accuracies attained were the same, having 98.84, all the hyperparameter combinations presented were placed in the first ranking. The row in bold format was the configuration utilized in training the prediction model.

For the k-NN, the distance functions used were Euclidean and Manhattan metrics, and the neighbors and weights hyperparameters were also tuned. The results of the hyperparameter optimization for k-NN are presented in Table 9.

In Table 9, 10 experimental results for the hyperparameter optimization of k-NN were displayed, describing the values for the metric, neighbors, weights hyperparameters, the attained accuracy for each combination, and the ranking. For k-NN, we found 3 hyperparameter combinations that obtained the highest score, which is 98.83. For the hyperparameter metric, the Manhattan distance function was listed for all the top 10 results. The values 9, 7 and 5 for the neighbors and “distance” as the value for the weights hyperparameter were the best configurations. For the weights hyperparameter, the value “uniform” can also be used and will also obtain a good accuracy of 98.14 to 98.75.

For the naïve Bayes algorithm, we tuned the var smoothing hyperparameter; the results are displayed in Table 10.

In Table 10, 10 experimental results for the hyperparameter optimization of NB were displayed, describing the values for the variance smoothing hyperparameter, the attained accuracy for each combination, and the ranking. Based on the results, we found that 2 variance smoothing alpha values of 0.1 and 0.123 obtained the highest score of 95.08.

We also examined the ANN regarding its performance in the experiment; the hyperparameters tuned were the number of hidden layers, activation function, solver, alpha, and learning rate. The results for the hyperparameter optimization for the ANN were displayed in Table 11.

In Table 11, 10 experimental results for the hyperparameter optimization of ANN are displayed, describing the values for the size of hidden layers, activation function, solver, alpha, and learning rate. For comparison, Table 11 displays the attained accuracy for each combination and the ranking. For ANN, we found 5 hyperparameter combinations that obtained the highest accuracy, which is 98.84. Hidden layers sizes of (50, 100, 50) and (50, 50, 50), activation function of relu and tanh, adam solver, alpha values of 0.0001 and 0.05, and the constant and adaptive learning rate were the most appropriate hyperparameter combinations to attain the highest possible accuracy of the ANN algorithm.

After the hyperparameter optimization process, we used the results in deciding what is the best configuration for each algorithm that will yield the highest possible accuracy. For the comparative analysis, the supervised machine learning algorithms using the best configurations were utilized in building a model that will predict the presence of COVID-19 in person. The developed models were evaluated using the training and testing dataset wherein the results were discussed in Section 3, which is the Results and Discussion section. Then, we used the serialization function to transform the developed model into a file that can be transmitted to other platforms. The joblib serializing package was installed, having the dump() and load() functions; this package has the capability of compiling a model into a file object, which is compatible and can be integrated in the next phase of this study, which is the development of a machine learning-based web application.

### 2.6. Machine Learning-Based Web Application Development

**Figure 6:**
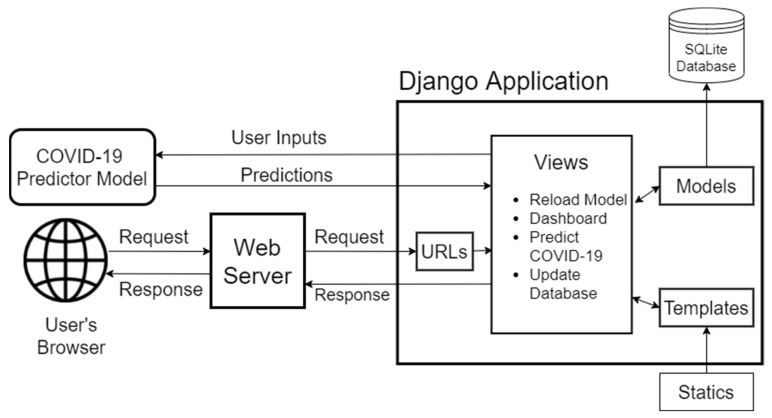
The overall process of the web application development phase.

To utilize the benefits of the developed COVID-19 prediction model, a web-based application must be developed to allow the users to input a new set of data to be predicted, which will be deployed later to be accessible for public use. The overall process of the web application development can be seen in Figure 6.

In Figure 6, the processes used to develop the web-based application using the Django Python-based framework were presented. The COVID-19 prediction model was used to predict the requests from the users which were collected and processed using Django’s Uniform Resource Locator (URL) patterns, views, models and templates. The results or the application’s response will be given back to the user containing the notification of being COVID-19 positive or negative.

#### 2.6.1. Django Python-Based Web Framework

Django is a Python based high level framework used in web development, which is simple, robust, and flexible [45]. Django can be described as a powerful tool to create applications that can be used easily by writing with less codes and provides a free administrator interface that allows authenticated users to add, update, and delete data. As presented in Figure 6, the Django application has four important modules to design: the URLs, views, models, and templates.

1. URLs

Django promotes a good URL design without displaying long lines of URL and file extensions such as .php or .asp [45]. To design URLs for an app, a Python module called “URLconf” needs to be created, which serves as a table of contents for the web application where the users can call for a particular page and returns error code 404 if the requested page was not found.

2. Views

A Django view is capable of returning a response, raising an exception such as error code 404, and giving the result of the functions or computations defined in the program. A Django view can collect data according to the defined parameters, load a web-page template, and display the web page according to the template together with the collected data. In views, the web-applications processes were defined such as the reloading of the COVID-19 prediction model, displaying of the dashboard, predicting COVID-19, prediction results notification, and the updating of the database. The COVID-19 prediction model discussed in Section 2 was also loaded in the views module using the joblib load() function. This process integrates the capability of the model to predict COVID-19 positive and COVID-19 negative samples by reconstructing a Python object from the serialized COVID-19 prediction model file.

3. Models

Django can be used with or without using a database, though it provides an object-relational mapper for systems that require a database layout, which can be described in Python codes [45]. We used the SQLite database, which is a lightweight disk-based database used in storing the data from the users, which can be used in the future as additional training samples to further improve the prediction rate of the COVID-19 prediction model.

4. Templates

The templates module is where the designed web-pages were located, which can be seen by the end users. Django has a good mechanism in handling the templates than can minimize the redundancy among the templates by using the benefits of “template inheritance”, where a base model can be developed that can be inherited by the rest of the templates [45]. In this way, the updating of the information and the design of the website can be done by just updating a single file. In the templates, Django also provides static file management to serve additional files used in web designing such as images, JavaScript (JS), or Cascading Style Sheets (CSS). Moreover, we make use of the Bootstrap to obtain professional-looking templates; currently, it is the world’s most popular front-end open-source toolkit used to quickly design and customize web and mobile responsive web pages [46].

From the user’s browser, a request of predicting the inputted data will then be sent to the web server, and the web server will give inputted data to the Django application for processing. The above-mentioned modules will work hand in hand to collect, process, and predict the submitted data using the COVID-19 prediction model loaded in the views module. The collected data together with the predicted result, either COVID-19 positive or negative, will be stored in the SQLite database. Then, a response will be made available to be transmitted to the user.

#### 2.6.2. Web-Based Application Workflow and the Developed System

**Figure 7:**
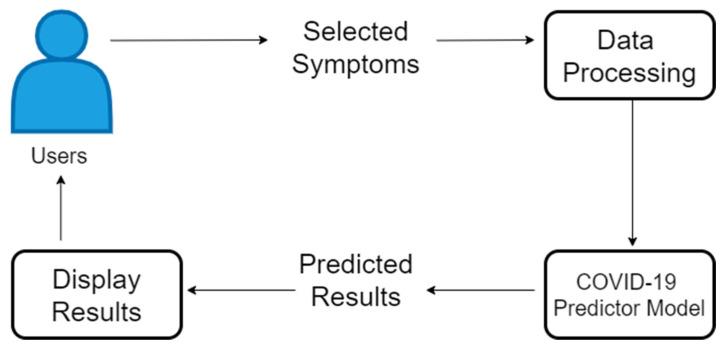
The workflow of the developed web-based application.

The workflow of the developed web-based application can be seen in Figure 7.

In Figure 7, the process starts from the user inputting the symptoms currently being experienced; then, the system will process the data to be ready for the prediction process, and then, the predicted results will be displayed in the user’s browser. The user of the application can access the application using a browser, in a desktop, mobile phone, or any device.

### 2.7. Deploying Django Services to Microsoft Azure Web Applications

**Figure 8:**
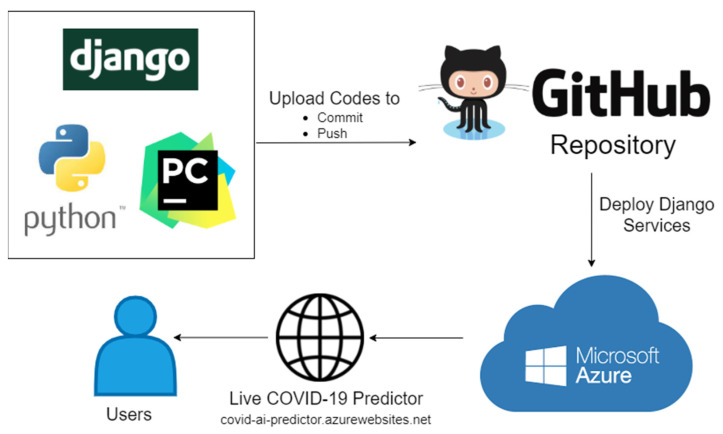
The process of deploying the web application in GitHub Repository and Microsoft Azure.

After the development of the machine learning-based web application, it is now ready for the deployment to make it available for public use. To deploy the web application, we used the GitHub Repository and Microsoft Azure. The process of deploying the web application is described in Figure 8.

In Figure 8, the process of deploying the web application in GitHub Repository and Microsoft Azure was displayed. PyCharm is an Integrated Development Environment (IDE) for Python programming language; it makes the coding easier, for it comes with smart code completion, code inspections, automatic error highlighting, debugging suggestions, and deployment capabilities. Moreover, PyCharm has first-class support for various web development technologies such as JavaScript, HTML/CSS, AngularJS, Node.js, and more, and it supports a number of web development frameworks such as Django [47]. We make use of the features of PyCharm IDE from the development of the web application using the Django framework, integration of the machine learning model, up to the deployment to GitHub Repository using the commit and push functionalities.

GitHub is popular, and it is considered as the world’s largest software development platform that hosts millions of repositories by providing cloud storage for source codes, and it supports all popular programming languages [48]. GitHub also provides a collaboration platform for its users to make code sharing, working with the same files, and merging projects easier. We utilized the commit and push features of GitHub, which is also available in PyCharm Professional to upload the prediction model and the source codes to the GitHub repository. The prediction model and the source codes are publicly accessible using the link provided in the Supplementary Materials section.

Microsoft developed Microsoft Azure, which is a cloud computing platform used specifically for building, deploying, and managing intelligent applications and services [49]. Upon logging in to Microsoft Azure, we created a web app resource and used covid-ai-predictor as the web application name and used Python as the runtime stack. To deploy and build code from GitHub, we logged into the GitHub account, chose to create a repository for the COVID-19 prediction application, and connected to the master branch. During the building and deployment, the requirements.txt file created using the “pip freeze” command was used in installing the dependencies of the project to allow the application to run smoothly.

## 3. Results and Discussion

The results of this study include two main subdivisions. The first one is the results of the comparative analysis for the developed prediction model presented in Section 3.1. The best prediction model was utilized in building a machine learning-based web application. The user interface screenshots of the developed web application can be seen in Section 3.2.

### 3.1. Results for Comparative Analysis

We used the hyperparameter optimization results shown in Table 6, Table 7, Table 8, Table 9, Table 10 and Table 11 to perform a comparative analysis to determine which supervised machine learning algorithm to use and its best hyperparameter configuration for training and testing the COVID-19 prediction model.

These results were indispensable in the comparative analysis process; we used these values as deciding factors in determining the most appropriate algorithm to be utilized in building a COVID-19 prediction model. The developed model’s performance evaluation result during the training process is summarized in Table 12.

In Table 12, the developed model’s performance evaluation results in the training dataset were displayed. Based on the results, the RF, SVM, k-NN, and ANN using tuned hyperparameters were the most appropriate supervised machine learning algorithms to be utilized in developing the COVID-19 prediction model. These three algorithms obtained the score of 98.84% accuracy, 100% sensitivity, 97.69% specificity, and 98.84% AUC applied in the COVID-19 Symptoms and Presence dataset. The confusion matrix results for the experiments performed during the model training are presented in Table 13, Table 14 and Table 15.

In Table 13, the confusion matrix result in the training dataset of the model using J48 DT was presented. Based on the prediction results, the 3068 samples that belong to the Negative class were successfully predicted. Whereas for the Positive class, among 3068 samples, the developed model successfully predicted 2982 samples, and the remaining 86 samples were classified as Negative.

In Table 14, the confusion matrix results of the models in the training dataset using RF, SVM, and k-NN algorithms are presented for these three algorithms, and the results obtained were the same. The 3068 samples that belong to Negative class were successfully predicted. Whereas for the Positive class, among 3068 samples, the developed model successfully predicted 2997 samples, and the remaining 71 samples were classified as Negative.

In Table 15, the confusion matrix result in the training dataset of the model using J48 DT was presented. For the 3068 samples that belong to the Negative class, 2967 were successfully predicted, but 101 samples were predicted as positive, which is the only algorithm that had misclassifications on the Negative class. For the Positive class, among 3068 samples, the developed model successfully predicted 2865 samples, but the remaining 203 were classified as Negative, which is the highest misclassification compared to the performance of the other algorithms.

After the training, the developed models were also evaluated using the test dataset, and the results of the model’s accuracy performance, sensitivity, specificity, and AUC are displayed in Table 16.

In Table 16, the developed model’s performance evaluation results in the test dataset were displayed. Based on the results, the RF, SVM, k-NN, and ANN using tuned hyperparameters were the most appropriate supervised machine learning algorithms to be utilized in developing the COVID-19 prediction model, using the same intuition as what we drew from the training dataset. These algorithms obtained the score of 98.75% accuracy, 100% sensitivity, 97.49% specificity, and 98.75% AUC applied in the devised testing dataset. The confusion matrix results for the experiments performed during the model testing are presented in Table 17, Table 18 and Table 19.

In Table 17, the confusion matrix results of the model using J48 DT in the test dataset are presented. The 1315 samples that belong to the Negative class were successfully predicted as Negative. Whereas for the Positive class, among 1315 samples, the developed model successfully predicted 1273 samples, and the remaining 42 samples were classified as Negative.

In Table 18, the confusion matrix results of the models using RF, SVM, k-NN, and ANN algorithms are presented. The 1315 samples that belong to the Negative class were successfully predicted as Negative. Whereas for the Positive class, among 1315 samples, the developed model successfully predicted 1282 samples as Positive, only 33 samples were classified as Negative.

In Table 19, the confusion matrix results in the training dataset of the model using J48 DT are presented. For the 1315 samples that belong to the Negative class, 1264 were successfully predicted, but 51 samples were predicted as positive. For the Positive class, among 3068 samples, the developed model successfully predicted 1233 samples, but the remaining 82 were classified as Negative.

Based on the results of the comparative analysis, it can be drawn that RF, SVM, k-NN, and ANN were the most appropriate algorithms to be considered in developing the COVID-19 prediction model outweighing the performance of the J48 DT and NB algorithms. The best configuration of the algorithms was also determined using the hyperparameter optimization grid search method and 10-fold cross-validation. The results obtained were used to achieve the highest possible performance of the models. For the hyperparameter optimization, RF, SVM, and ANN yielded the best score, 98.84%, while the k-NN obtained 98.83%. In using the training dataset, the RF, SVM, k-NN, and ANN algorithms yielded 98.84% accuracy, 100% sensitivity, 97.69%, specificity, and 98.84% AUC.

### 3.2. The Developed Machine Learning-Based Web Application

**Figure 9:**
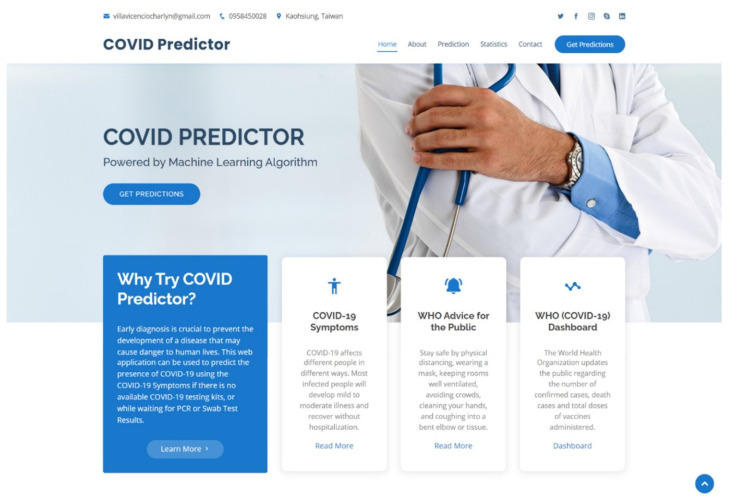
The dashboard, home, or the landing page when a user accessed the web-based application using a browser.

The dashboard of the web application is presented in Figure 9.

In Figure 9, the email address, contact number, location, and social media links of the researcher were displayed for communication purposes. There were several links that the user could choose: Home, About, Prediction, Statistics, Contact, and Get Predictions. The About portion displays information about the COVID-19 prediction model and informative links that redirect to the WHO’s website, which can be accessed by clicking the “Read More”, including a list of COVID-19 symptoms, the WHO’s advice to the public, and the WHO’s COVID-19 website’s dashboard. The WHO’s dashboard consists of an interactive map in a color-coded scheme regarding the number of cases, a country can be selected in the map, and information such as confirmed cases, deaths, and vaccine doses’ administered statistics will be presented. Moreover, bar charts of confirmed cases and deaths were presented that offer convenience of data analysis through time.

**Figure 10:**
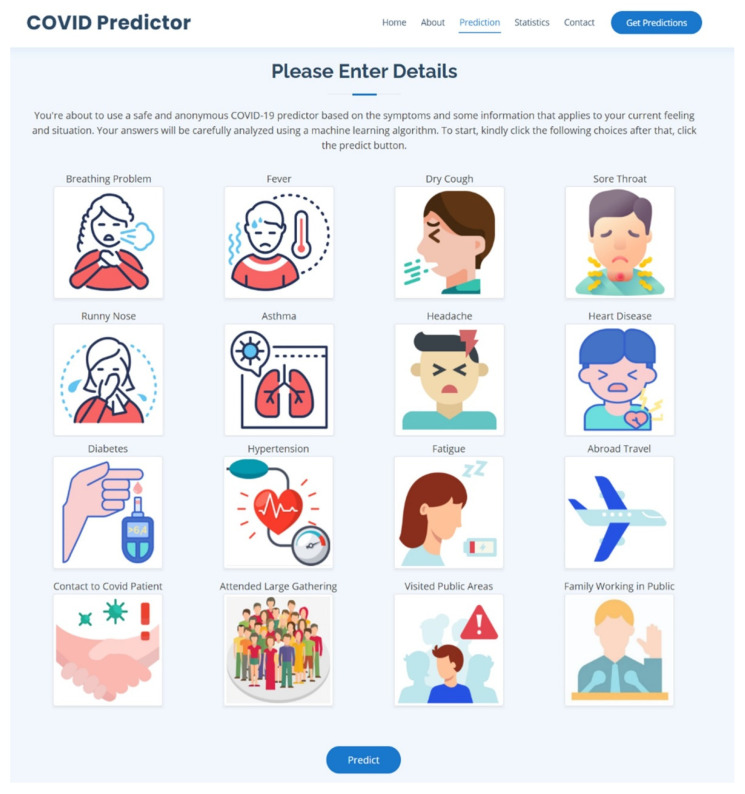
The COVID-19 Prediction section.

The next section is the main part of the web application, which is the COVID-19 Prediction; it can be accessed by clicking the Get Predictions button on the navigation bar and the home page and by scrolling down the web page. The COVID-19 Prediction section can be seen in Figure 10.

**Figure 11:**
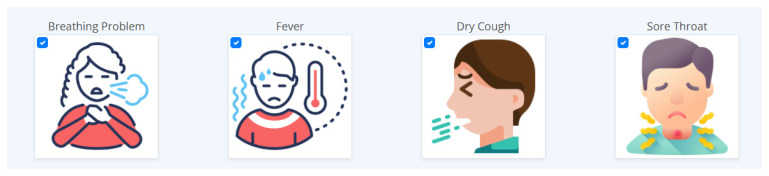
The activated checkbox of the selected symptoms.

In Figure 10, the COVID-19 Prediction section is presented, and there were 16 symptoms presented, including breathing problems, fever, dry cough, sore throat, runny nose, asthma, headache, heart disease, diabetes, hypertension, fatigue, abroad travel, contact with COVID-19 patient, attended a large gathering, visited public areas, and family working in public. This application is capable of allowing the user to choose the symptoms currently being experienced by clicking on it, and the user can click more than one symptom. When a user clicks on a symptom, the respective checkbox for it will be activated, and the selected symptoms representation is displayed in Figure 11.

**Figure 12:**
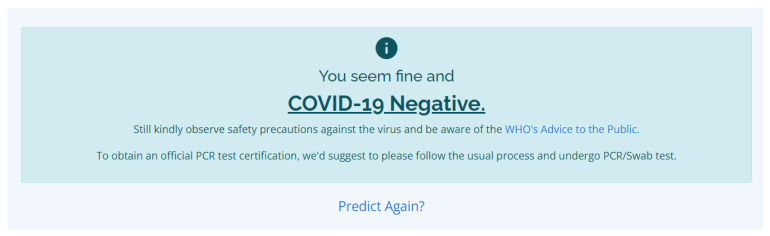
The notification for the COVID-19 Negative result.

In Figure 11, the user can select multiple symptoms, all of the symptoms, or none at all; in case the user accidentally clicked on an inappropriate symptom, the user can deselect it by simply clicking on the symptom again, and the checkbox will go back to its original state. After selecting all of the applicable symptoms or the symptoms currently being experienced by the user, the predict button can be clicked, which is an indication that the application will now process the inputted data, which will be loaded into the machine learning model for prediction. Afterwards, when the result is ready, one of the following notifications will be presented on the user’s device. The COVID-19 Negative result notification can be seen in Figure 12.

**Figure 13:**
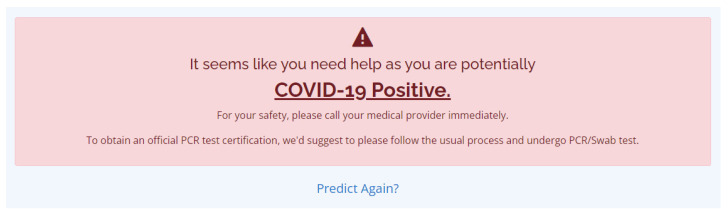
The notification for the COVID-19 Positive result.

In Figure 12, we used the phrase “You seem fine and COVID-19 Negative”, which indicates that the developed COVID-19 classifier’s prediction regarding the inputted symptoms by the user is COVID-19 Negative. Even though the result is negative, the user is still expected to observe safety precautions against the virus and be informed about the WHO’s advice to the public. It is also indicated that the COVID-19 prediction results are unofficial, and to obtain the official PCR test certification, it is suggested to follow the usual process and undergo PCR testing. Another notification that the user may possible receive is the COVID-19 positive notification, and the message can be seen in Figure 13.

In Figure 13, we opted to use a gentle phrase “It seems like you need help as you are potentially COVID-19 Positive” which indicates that the classifier’s prediction with respect to the selected symptoms by the user is COVID-19 Positive. The notification highly suggests to call a medical provider immediately to seek proper medical attention; this result is unofficial, and it is necessary to undergo PCR testing as soon as possible to obtain official results and to promote the safety of the user and other people. Lastly, a predict-again hyperlink was provided to enable users who wish to use the COVID-19 prediction again.

**Figure 14:**
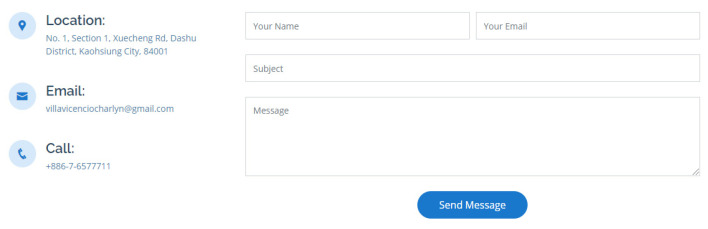
The contact form of the website.

A contact section was also provided in the last portion of the web application; it displays the researcher’s email, and the university contact number. Moreover, a contact form was made available that can be used to contact the researcher and improve the application by sharing messages, comments, suggestions, or report bugs encountered in the system. The contact section can be seen in Figure 14.

**Figure 15:**
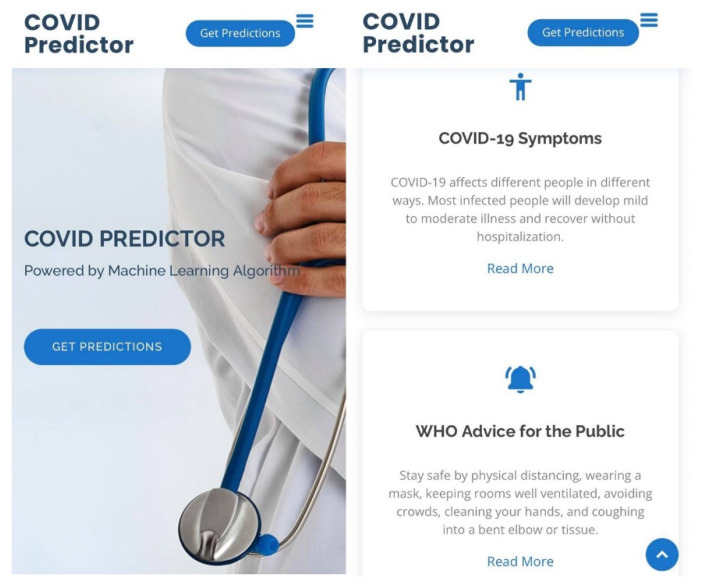
This is the dashboard, home, and about viewed in a mobile browser.

**Figure 16:**
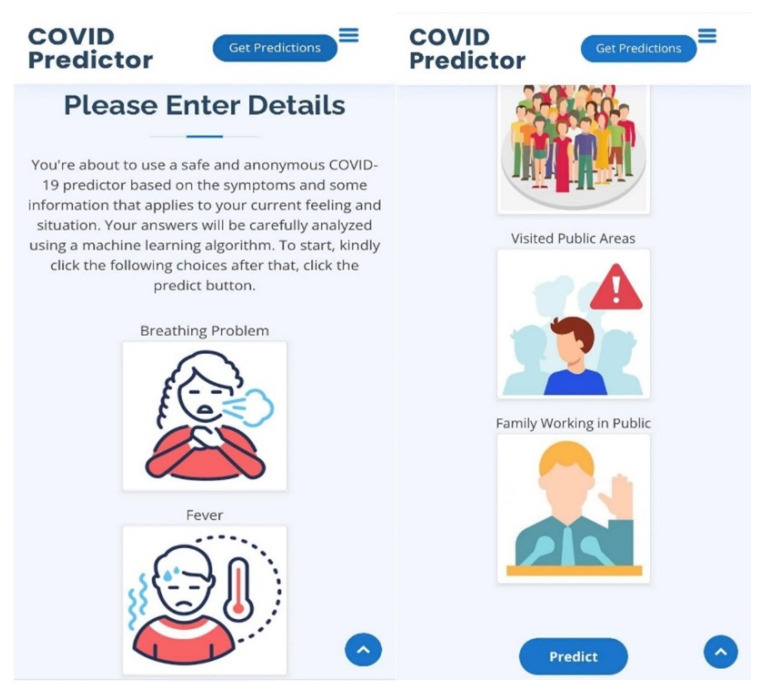
The COVID-19 prediction section responsive view section using a mobile device.

**Figure 17:**
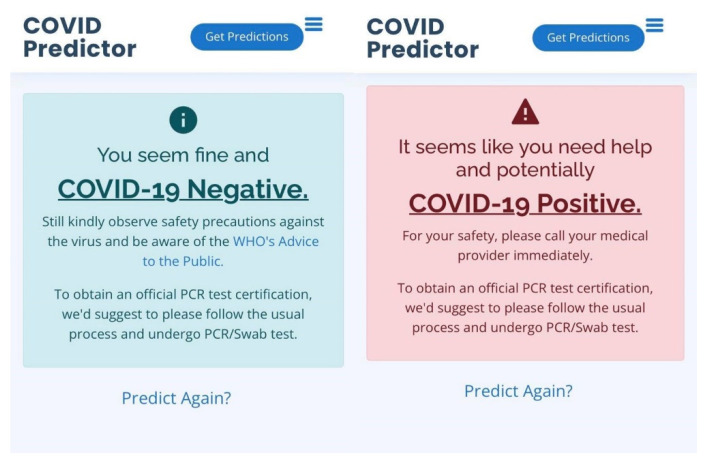
The COVID-19 negative and positive notifications viewed in a mobile device.

In Figure 14, the contact form is presented, email address of the developer, and university contact number. The web application was made responsive to any devices; hence, the mobile screenshots can be seen in Figure 15, Figure 16 and Figure 17.

In Figure 16, the COVID-19 prediction section viewed in a mobile device was presented; this time, the users can just tap on the appropriate symptoms currently being experienced and tap on the Predict button to start the prediction. Multiple selection is allowed in case the user is experiencing a combination of symptoms.

These notifications will be given to the user. The message indicates that the results were unofficial, and the user needs to follow the usual process to undergo PCR or swab testing to obtain official results and certification.

## 4. Conclusions

This study aimed to build a COVID-19 prediction model by applying supervised machine learning algorithms. J48 decision tree, random forest, support vector machine, k-nearest neighbors, naïve Bayes, and artificial neural network were the algorithms used in this study. The most appropriate configurations were successfully determined through hyperparameter optimization and 10-fold cross-validation. A comparative analysis was conducted by evaluating each model’s performance in terms of its accuracy, sensitivity, specificity, and area under ROC curve, using the Google colab research. The results show that random forest, support vector machine, k-nearest neighbor, and artificial neural network were the best algorithms to be utilized in building the COVID-19 prediction model. These algorithms yielded 98.84% accuracy, 100% sensitivity, 97.69% specificity, and 98.84% area under the ROC curve score. The developed models were successfully integrated in the development of a machine learning based web application using the Django Framework, which was written in Python programming language using the PyCharm professional IDE. The source codes were deployed in the GitHub repository, and Microsoft Azure Web App service features were used to make the web application accessible online. The output of this study is a COVID-19 prediction application that allows the user to input appropriate symptoms currently being experienced and automatically receive a notification indicating the result of the classifier: either COVID-19 positive or negative. This study can serve as an additional tool for people who wish to manage their health status with regard to COVID-19 in a real-time manner without the need for laboratory tests.

## Figures and Tables

**Table 1:** COVID-19 Prediction Models.

References	COVID-19 Prediction	Predictors Used	Algorithms	Accuracy
[5]	COVID-19 prediction using laboratory findings	Laboratory findings	Convolutional Neural Network (CNN)	76.00%
[6]	Prediction of the severity of COVID-19 using blood test results	Blood test results	Multiple-criteria decision-making (MCDM)	82.00%
[7]	COVID-19 prediction using X-ray images	X-ray images	Visual Geometry Group (VGG16)	98.60%
[8]	Prediction of the mortality of a patient	Blood samples	Extreme Gradient Boosting (XGBoost)	90.00%
[9]	COVID-19 prediction using CT images	Computed tomography (CT) images	DenseNet201 Feature Pyramid Network (FPN)	98.96%
[10]	COVID-19 prediction using X-ray images	X-ray images	Deep Neural Network (DNN)	90.50%
Proposed Method	COVID-19 prediction based on symptoms	COVID-19 symptoms	Supervised Machine Learning Algorithms	98.84%

**Table 2:** COVID-19 predictors and descriptions of the COVID-19 symptoms and presence dataset [3].

Attribute Name	Description
Breathing problems	Experiencing shortness of breath, having trouble breathing
Fever	Temperature is above normal
Dry cough	Continuous coughing without phlegm
Sore throat	Experiencing pain, scratchiness, and irritation in the throat
Runny nose	Having a nasal drainage such as thin fluids, thick mucus, or frequent sneezing
Asthma	Diagnosed with asthma
Chronic lung disease	Diagnosed with lung disease
Headache	Pain in the head or in a certain part of the head
Heart disease	Diagnosed with cardiovascular disease
Diabetes	Diagnosed with diabetes
Hypertension	Having a high blood pressure
Fatigue	Constantly feeling tired and weak
Gastrointestinal	Digestive system problems
Abroad travel	Has recent travel history
Contact with COVID-19 patient	Physical contact with COVID-19 positive people
Attended large gathering	The person or anyone from the family recently attended a mass gathering.
Visited public exposed places	Recently visited malls, temples, and other public places
Family working in public exposed places	Relatives are working in a market, hospital, or crowded place.
Wearing masks	Wearing face masks properly
Sanitation from market	Disinfecting products bought from market before using
COVID-19	The presence of COVID-19

**Table 3:** The correlation of predictors to COVID-19 attributes.

Attribute Name	Correlation Value
**Sore Throat**	**0.503**
Dry Cough	0.464
Abroad Travel	0.444
Breathing Problems	0.444
Attended a Large Gathering	0.390
Contact with COVID-19 Patient	0.357
Fever	0.353
Family Working in Public	0.160
Visited Public Exposed Places	0.112
Hypertension	0.103
Asthma	0.090
Diabetes	0.041
Heart Disease	0.027
Gastrointestinal	−0.003
Runny Nose	−0.006
Headache	−0.028
Fatigue	−0.044
Chronic Lung Disease	−0.057

**Table 4:** VIF of predictors in the dataset.

Attribute Name	VIF
**Dry cough**	**5.38**
Fever	4.95
Sore throat	4.79
Breathing problems	3.48
Contact to COVID patient	2.28
Abroad travel	2.19
Runny nose	2.17
Attended a large gathering	2.12
Visited public	2.09
Headache	2.06
Hypertension	2.01
Asthma	1.95
Diabetes	1.94
Fatigue	1.93
Gastrointestinal	1.89
Heart disease	1.85
Family working in public	1.84
Chronic lung disease	1.76

**Table 5:** Symptoms of COVID-19 [22].

Most Common	Less Common	Serious
Fever	Sore throat	Difficulty in breathing
Cough	Headache, aches and pains	Shortness of breath
Fatigue	Diarrhea	Loss of speech or mobility
Loss of smell	Rashes/discoloration of skin, fingers or toes	Confusion
Loss of taste	Red or irritated eyes	Chest pain

**Table 6:** J48 DT algorithm hyperparameter optimization results.

No	Criterion	Min Samples in the Node	Maximum Depth	Accuracy	Ranking
**1**	**Gini**	**20**	**5**	**98.52**	**1**
2	Gini	10	5	98.52	1
3	entropy	20	5	98.48	3
4	entropy	10	5	98.48	3
5	entropy	20	10	98.27	5
6	entropy	10	10	98.27	5
7	Gini	20	10	98.16	7
8	Gini	10	10	98.16	7
9	entropy	20	20	97.08	9
10	entropy	10	20	97.08	9

**Table 7:** Random forest algorithm hyperparameter optimization results.

No	Criterion	Max Depth	Min Samples in the Node	No. of Estimators	Bootstrap	Accuracy	Ranking
**1**	**Gini**	**20**	**5**	**200**	**False**	**98.75**	**1**
2	entropy	20	5	300	True	98.73	2
3	Gini	20	5	300	True	98.71	3
4	entropy	20	5	300	False	98.71	4
5	entropy	20	5	200	True	98.70	5
6	entropy	20	5	100	True	98.70	5
7	Gini	20	5	100	False	98.70	7
8	Gini	10	5	300	False	98.70	7
9	Gini	20	5	100	True	98.70	9
10	Gini	10	5	200	False	98.70	9

**Table 8:** Support Vector Machine algorithm hyperparameter optimization results.

No	C	Degree	Gamma	Kernel	Accuracy	Ranking
**1**	**5**	**-**	**1**	**Radial Basis Function**	**98.84**	**1**
2	1	2	1	Polynomial	98.84	1
3	10	-	1	Radial Basis Function	98.84	1
4	2	-	1	Radial Basis Function	98.84	1
5	10	-	0.1	Radial Basis Function	98.84	1
6	5	-	0.1	Radial Basis Function	98.84	1
7	10	2	1	Polynomial	98.84	1
8	5	-	0.1	Radial Basis Function	98.84	1
9	10	-	1	Radial Basis Function	98.84	1
10	1	3	1	Polynomial	98.84	1

**Table 9:** K-nearest neighbors algorithm hyperparameter optimization results.

No	Metric	Neighbors	Weights	Accuracy	Ranking
**1**	**Manhattan**	**9**	**distance**	**98.83**	**1**
2	Manhattan	7	distance	98.83	1
3	Manhattan	5	distance	98.83	1
4	Manhattan	11	distance	98.79	4
5	Manhattan	3	distance	98.78	5
6	Manhattan	13	distance	98.76	6
7	Manhattan	3	uniform	98.75	7
8	Manhattan	5	uniform	98.58	8
9	Manhattan	7	uniform	98.39	9
10	Manhattan	9	uniform	98.14	10

**Table 10:** Naïve Bayes algorithm hyperparameter optimization results.

No	Variance Smoothing	Accuracy	Ranking
**1**	**0.1**	**95.08**	**1**
2	0.123	95.08	1
3	0.231	95.06	3
4	0.043	95.05	4
5	0.187	95.05	5
6	0.152	95.05	5
7	0.053	95.03	7
8	0.066	95.03	8
9	0.081	95.01	9
10	0.285	95.01	10

**Table 11:** Artificial neural network algorithm hyperparameter optimization results.

No	Hidden Layer Sizes	Activation	Solver	Alpha	Learning Rate	Accuracy	Ranking
**1**	**(50, 100, 50)**	**relu**	**adam**	**0.0001**	**constant**	**98.84**	**1**
2	(50, 100, 50)	tanh	adam	0.0001	constant	98.84	1
3	(50, 100, 50)	relu	adam	0.05	constant	98.84	1
4	(50, 100, 50)	relu	adam	0.0001	adaptive	98.84	1
5	(50, 50, 50)	tanh	adam	0.05	constant	98.84	1
6	(50, 50, 50)	relu	adam	0.0001	adaptive	98.83	6
7	(50, 50, 50)	relu	adam	0.05	adaptive	98.81	7
8	(50, 50, 50)	tanh	adam	0.05	adaptive	98.79	8
9	(50, 100, 50)	tanh	adam	0.05	constant	98.78	9
10	(50, 50, 50)	relu	adam	0.0001	constant	98.76	10

**Table 12:** Developed model’s performance evaluation result (training).

Algorithm	Accuracy	Sensitivity	Specificity	AUC
J48 DT	98.60	100.00	97.20	98.60
**RF**	**98.84**	**100.00**	**97.69**	**98.84**
**SVM**	**98.84**	**100.00**	**97.69**	**98.84**
**k-NN**	**98.84**	**100.00**	**97.69**	**98.84**
NB	95.05	96.71	93.38	95.05
**ANN**	**98.84**	**100.00**	**97.69**	**98.84**

**Table 13:** Confusion matrix for J48 DT model (training).

Actual	Predicted Negative	Predicted Positive
Negative	3068	0
Positive	86	2982

**Table 14:** Confusion matrix for RF, SVM, k-NN, and ANN models (training).

Actual	Predicted Negative	Predicted Positive
Negative	3068	0
Positive	71	2997

**Table 15:** Confusion matrix for NB model (training).

Actual	Predicted Negative	Predicted Positive
Negative	2967	101
Positive	203	2865

**Table 16:** Developed model’s performance evaluation result (training).

Algorithm	Accuracy	Sensitivity	Specificity	AUC
J48 DT	98.40	100.00	96.81	98.40
**RF**	**98.75**	**100.00**	**97.49**	**98.75**
**SVM**	**98.75**	**100.00**	**97.49**	**98.75**
**K-NN**	**98.75**	**100.00**	**97.49**	**98.75**
NB	94.94	96.12	93.76	94.94
**ANN**	**98.75**	**100.00**	**97.49**	**98.75**

**Table 17:** Confusion matrix for J48 DT model (testing).

Actual	Predicted Negative	Predicted Positive
Negative	1315	0
Positive	42	1273

**Table 18:** Confusion matrix for RF, SVM, k-NN, and ANN models (testing).

Actual	Predicted Negative	Predicted Positive
Negative	1315	0
Positive	33	1282

**Table 19:** Confusion matrix for NB model (testing).

Actual	Predicted Negative	Predicted Positive
Negative	1264	51
Positive	82	1233

## Data Availability

The dataset utilized in this study is available at https://www.kaggle.com/hemanthhari/symptoms-and-covid-presence (accessed on 30 June 2021). The prediction model and the source codes used to develop the machine learning-based web application is publicly accessible through this GitHub repository https://github.com/dearcharlyn/covid_predictor (accessed on 30 June 2021).

## Supplementary Materials

The prediction model and the source codes used to develop the machine learning-based web application can be downloaded at https://github.com/dearcharlyn/covid_predictor (accessed on 23 March 2022).
